# Clinical significance of preoperative neutrophil‐lymphocyte ratio and platelet‐lymphocyte ratio in the prognosis of resected early‐stage patients with non‐small cell lung cancer: A meta‐analysis

**DOI:** 10.1002/cam4.5505

**Published:** 2022-12-08

**Authors:** Weibo Cao, Haochuan Yu, Shuai Zhu, Xi Lei, Tong Li, Fan Ren, Ning Zhou, Quanying Tang, Lingling Zu, Song Xu

**Affiliations:** ^1^ Department of Lung Cancer Surgery Lung Cancer Institute, Tianjin Medical University General Hospital Tianjin China; ^2^ Tianjin Key Laboratory of Lung Cancer Metastasis and Tumor Microenvironment Lung Cancer Institute, Tianjin Medical University General Hospital Tianjin China

**Keywords:** meta‐analysis, neutrophil‐lymphocyte ratio, non‐small cell lung cancer, operation, platelet‐lymphocyte ratio, prognosis

## Abstract

**Background:**

Poor prognosis is linked to peripheral blood levels of preoperative platelet‐lymphocyte ratio (PLR) and neutrophil‐lymphocyte ratio (NLR) in many advanced cancers. Nevertheless, whether the correlation exists in resected early‐stage cases with non‐small cell lung cancer (NSCLC) stays controversial. Consequently, we performed a meta‐analysis to explore the preoperative NLR and PLR's prognostic significance in early‐stage patients with NSCLC undergoing curative surgery.

**Methods:**

Relevant studies that validated the link between preoperative NLR or PLR and survival results were found via the proceeding databases: PubMed, Embase, Cochrane Library, and Web of Science. The merged 95% confidence interval (CI) and hazard ratio (HR) was employed to validate the link between the NLR or PLR's index and overall survival (OS) and disease‐free survival (DFS) in resected NSCLC cases. We used sensitivity and subgroup analyses to assess the studies' heterogeneity.

**Results:**

An overall of 21 studies were attributed to the meta‐analysis. The findings indicated that great preoperative NLR was considerably correlated with poor DFS (HR = 1.58, 95% CI: 1.37–1.82, *p* < 0.001) and poor OS (HR = 1.51, 95% CI: 1.33–1.72, *p* < 0.001), respectively. Subgroup analyses were in line with the pooled findings. In aspect of PLR, raised PLR was indicative of inferior DFS (HR = 1.28, 95% CI: 1.04–1.58, *p* = 0.021) and OS (HR = 1.37, 95% CI: 1.18–1.60, *p* < 0.001). In the subgroup analyses between PLR and DFS, only subgroups with a sample size <300 (HR = 1.67, 95% CI: 1.15–2.43, *p* = 0.008) and TNM staging of mixed (I‐II) (HR = 1.47, 95% CI: 1.04–2.07, *p* = 0.028) showed that the link between high PLR and poor DFS was significant.

**Conclusions:**

Preoperative elevated NLR and PLR may act as prognostic biomarkers in resected early‐stage NSCLC cases and are therefore valuable for guiding postoperative adjuvant treatment.

## Graphical abstract

The meta‐analysis investigated the prognostic significance of preoperative NLR and PLR in early‐stage patients with NSCLC undergoing curative surgery. A total of 21 studies were enrolled in the metaanalysis. Summarizing the available data from the included studies, our results confirmed that high preoperative NLR and PLR were significantly associated with inferior DFS and OS. Therefore, the levels of preoperative NLR and PLR may serve as prognostic biomarkers in the clinic and are valuable measures for guiding postoperative adjuvant treatment.

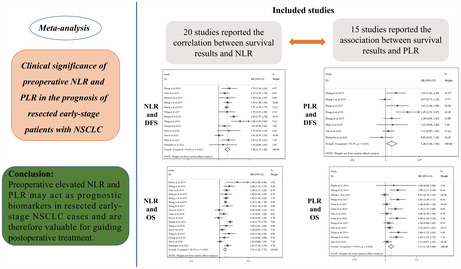

## 1 INTRODUCTION

Non‐small cell lung cancer (NSCLC), which makes up about 85% of lung malignancies, is the most repeatedly diagnosed cancer globally and the leading mortality reason among all other cancers.^1^ Notwithstanding the recent rapid advancements in molecular therapeutic strategies and immunotherapy, surgery is still the go‐to treatment for people with early‐stage NSCLC. However, when contrasted to thoracotomy, minimally invasive surgery (MIS), including video‐assisted thoracoscopic surgery (VATS), can significantly reduce the time patients need to stay in the hospital and the number of postoperative complications they experience.^2^, ^3^

Furthermore, the tumor‐node‐metastasis (TNM) classification system can be utilized to some extent for determining treatment strategy and prognostic evaluation in patients with NSCLC.^4^, ^5^ This is valuable for clinicians, for whom the prognosis of cases with lung cancer has always been the focus. Nevertheless, patients with similar TNM staging continuously have different clinic survival outcomes.

Due to accumulating studies, it has become increasingly evident that inflammation can affect the progression and invasion of lung cancer.^6^, ^7^ Systematic inflammatory cells, along with their calculated ratios, including platelet‐lymphocyte ratio (PLR), lymphocyte‐monocyte ratio (LMR), and neutrophil‐lymphocyte ratio (NLR), act as independent factors which influence the cases' prognosis with different cancers, involving lung,^8^ breast,^9^ and nasopharyngeal malignancies.^10^ A published meta‐analysis, involving 244 studies of cases with resected pan‐cancers, demonstrated that a close association exists between raised NLR/PLR and poor OS or cancer‐specific survival (CSS) and between raised LMR and poor OS.^11^ Another meta‐analysis, including cases with NSCLC medicated with immune checkpoint inhibitors (ICIs), also indicated that raised PLR and NLR were related to inferior progression‐free survival (PFS) and OS.^12^ Nevertheless, the link between NLR/PLR and survival outcomes in cases with resected NSCLC stays indefinite. Therefore, a comprehensive meta‐analysis is warranted to validate the NLR/PLR's prognostic significance in patients with NSCLC.

Accordingly, this meta‐analysis includes numerous relevant studies to explore the link between peripheral blood NLR/PLR and survival results in resected early‐stage NSCLC cases.

## 2 MATERIALS AND METHODS

### 2.1 Search strategy

We searched the literature from the four following databases: PubMed, Embase, Cochrane Library, and Web of Science. The primary deadline for literature retrieval was March 2022. The search strategy was performed using the following terms: (“Lung neoplasms” OR “pulmonary neoplasms” OR “lung cancer” OR “Carcinoma, Non‐Small‐Cell Lung” OR “non‐small cell lung cancer” OR “NSCLC”) AND (“Surgical Procedures, Operative” OR “surgery” OR “operative therapy” OR “operation” OR “operative procedures” OR “invasive procedures”) AND ((“neutrophil lymphocyte ratio” OR “neutrophil‐to‐lymphocyte ratio” OR “NLR”) OR (“platelet lymphocyte ratio” OR “platelet‐to‐lymphocyte ratio” OR “PLR”)). The specific retrieval strategy can be found in Supplementary Text S1.

### 2.2 Exclusion and inclusion criteria

Studies gathering the following requirements were involved: (a) populations involving early‐stage NSCLC patients; (b) populations of NSCLC patients for whom curative surgical resection was performed; (c) the link between NLR/PLR and prognostic indicators of OS and/or disease‐free survival (DFS) were investigated; (d) the 95% confidence interval (CI) and hazard ratio (HR) could be obtained from the original studies; (e) the literature was written in English.

The exclusion criteria were as follows: (a) reviews, case reports, abstracts, letters, and expert opinions; (b) populations of patients with other primary tumors; (c) surgical resection was not performed; (d) the studies had insufficient data to conclude on the HR and 95% CI; (e) literature with Newcastle‐Ottawa Scale (NOS) scores <6; (f) the literature was not published in English.

### 2.3 Literature's data extraction and quality validation

Two reviewers (Weibo Cao and Haochuan Yu) independently extracted relevant data and evaluated the studies' quality. Any disagreements were then resolved through discussion and consensus.

The first author's name, the year of publication, study design, country of origin, study design, sample size, pathological category of lung cancer, TNM, median follow‐up (months), NLR/PLR cut‐off values, and survival data (DFS and/or OS) were all extracted from each study. When both multivariate and univariate analyses were run, the multivariate analysis's HRs were extracted first. Our way of determining cut‐off values for subgroup analysis was exploited according to the median value from all of NLR or PLR cut‐off in the studies recruited in this meta‐analysis.

We evaluated the literature's quality involved according to the scoring system of the NOS. The NOS contains three aspects: patient selection, comparability, and outcome assessment. Literature with scores of ≥6 was considered of high quality. Studies with lower scores were regarded as low quality and therefore excluded from the analysis.

### 2.4 Statistical analysis

Higgins I^2^ and Cochran's Q test statistics were utilized to evaluate the studies' heterogeneity. *p* ≤ 0.10 or I^2^ ≥ 50% were significant heterogeneity indicative, and in such cases, a random‐effect model was utilized. Conversely, a fixed‐effect model was utilized to evaluate studies that did not present significant heterogeneity. Subgroup analyses dependent on the sample size, TNM, and cut‐off values were also performed to investigate potential factors influencing the NLR/PLR's prognostic significance. Sensitivity analyses were then performed to determine any heterogeneity's source and assess the results' stability. Publication bias assessment was conducted via Funnel plots and Egger's test, in which *p* < 0.05 was deemed statistically significant. All statistical analyses were done utilizing STATA 15.0 (Stata Corporation, College Station, TX).

## 3 RESULTS

### 3.1 Literature search and study characteristics

**FIGURE 1:**
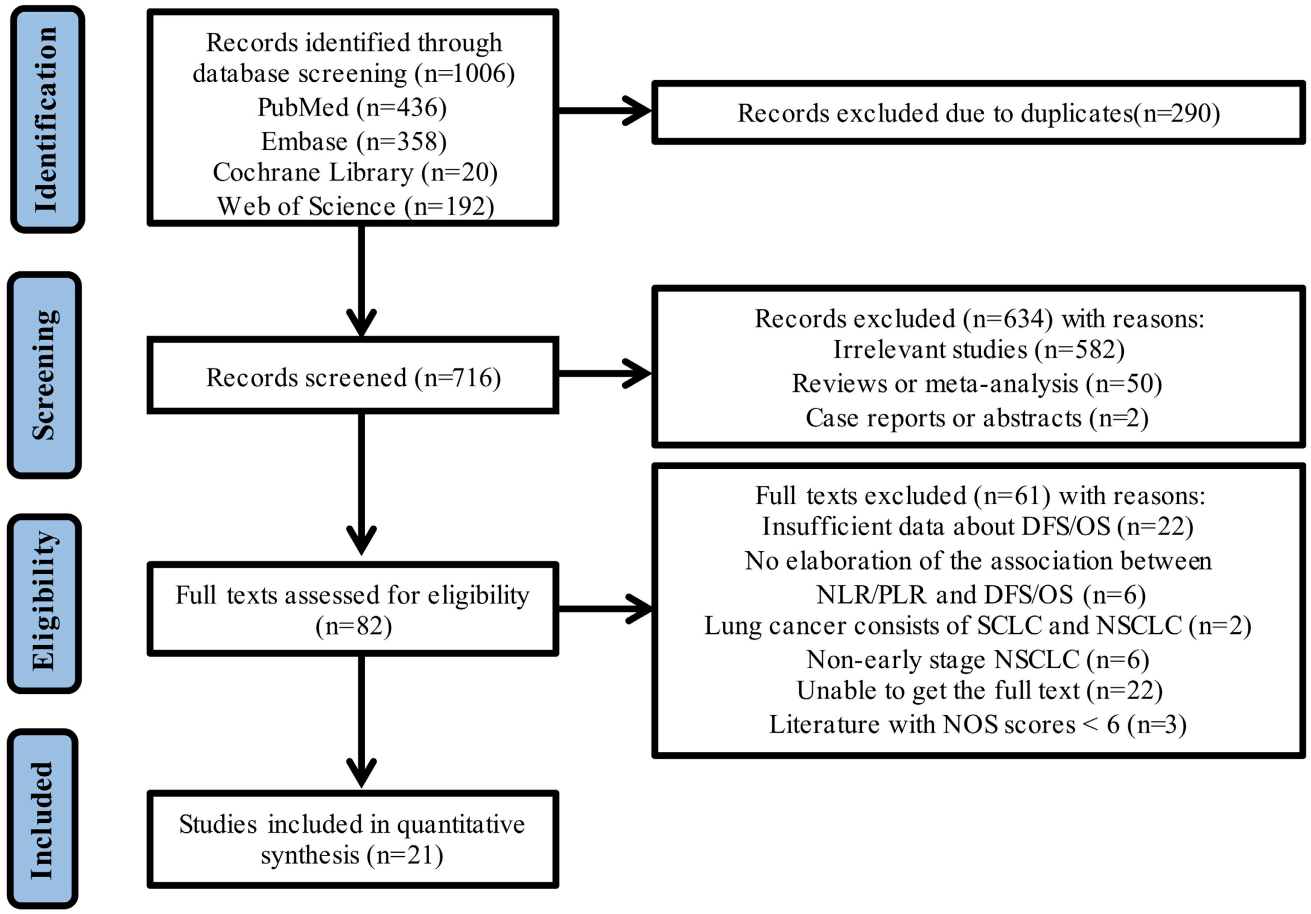
The flow diagram of literature selection.

As per the search approach, 1006 studies were initially identified, of which 290 were ruled out owing to duplication. By screening literature titles and abstracts, a further 634 studies were excluded based upon the exclusion criteria, resulting in 82 full‐text studies. Of these, 21 studies met the inclusion criteria and were subsequently involved in the meta‐analysis.^13^, ^14^, ^15^, ^16^, ^17^, ^18^, ^19^, ^20^, ^21^, ^22^, ^23^, ^24^, ^25^, ^26^, ^27^, ^28^, ^29^, ^30^, ^31^, ^32^, ^33^ The literature selection process is outlined in Figure 1.

Table 1 lists the studies' primary traits that made up the meta‐analysis. In conclusion, case sample sizes in the studies ranged from 134 to 2027, and the studies were all published between 2014 and 2021. Out of the included studies, six focused on NLR only, one concentrated on PLR only, and 14 evaluated both NLR and PLR. We had 20 NLR studies with 13,915 lung cancer cases and 15 PLR studies with 7484 lung cancer cases in the meta‐analysis. NOS scores for the quality of the involved literature varied from 6 to 9, demonstrating a high quality of literature among the 21 studies. Quality assessment of involved studies can be found in Supplementary Table  S1.

**TABLE 1:** Main characteristics of the studies included in the meta‐analysis

Author	Year	Region	Study design	Sample size	Histology	TNM	Postoperative adjuvant therapy	Median follow‐up (months)	Prognostic value	NLR cut‐off	PLR cut‐off	Outcomes
Pinato et al.	2014	Europe	P	220	NSCLC	I‐III	7% adjuvant CT; 7% adjuvant RT; 4% adjuvant CRT	NR	NLR, PLR	5	300	DFS, OS
Zhang et al.	2014	China	R	400	NSCLC	I‐II	NP	46	NLR, PLR	3.3	171	DFS, OS
Choi et al.	2015	America	R	1139	NSCLC	I‐III	25% adjuvant CT; 15% adjuvant RT	NR	NLR	5	NR	DFS, OS
Shimizu et al.	2015	Japan	R	334	NSCLC	I‐III	NR	32	NLR	2.5	NR	DFS, OS
Zhang 1 et al.	2015	China	R	678	NSCLC	I‐III	Adjuvant therapy^a^	43.5	NLR, PLR	2.3	106	DFS, OS
Zhang 2 et al.	2015	China	R	1238	NSCLC	I‐III	Adjuvant therapy^a^	45	NLR	2.3	NR	DFS, OS
Wang et al.	2017	China	R	134	SCC	I‐III	58% adjuvant CT	22	NLR, PLR	2.16	145	DFS, OS
Yuan et al.	2017	China	R	1466	NSCLC	I‐III	44% adjuvant CT	69.9	NLR, PLR	2.06	204	OS
Chen et al.	2018	China	R	577	NSCLC	I	16% adjuvant CT	93.77	NLR, PLR	3.13	81.07	OS
Gao et al.	2018	China	R	410	NSCLC	I‐III	NR	54	NLR, PLR	1.90	108.8	OS
Huang et al.	2018	China	R	589	NSCLC	I‐III	NR	44	NLR	2.3	NR	DFS, OS
Toda et al.	2018	Japan	R	327	NSCLC	I‐III	24% adjuvant CT	NR	PLR	NR	162	DFS, OS
Wang et al.	2018	China	R	952	NSCLC	I‐III	51% adjuvant CT	40	NLR, PLR	3.1	170.58	OS
Guo et al.	2019	China	R	569	NSCLC	I‐III	NR	60.3	NLR, PLR	1.74	88.7	OS
Wang et al.	2019	China	R	261	NSCLC	I‐III	NR	38	NLR, PLR	2.12	92.9	DFS, OS
Huang et al.	2019	China	R	254	NSCLC	I‐III	54% adjuvant CT; 7% adjuvant RT	48	NLR, PLR	3.18	122	DFS, OS
Shoji et al.	2020	Japan	R	311	NSCLC	I	NP	63	NLR, PLR	1.5	184	DFS
Yan et al.	2020	China	R	538	NSCLC	I‐III	58% adjuvant CT	54	NLR, PLR	2.35	150.95	DFS, OS
Shen et al.	2021	China	R	1431	AD	I	13% adjuvant CT	63	NLR	2.606	NR	DFS
Watanabe et al.	2021	Japan	R	387	NSCLC	I‐III	28% adjuvant CT	39.2	NLR, PLR	2.90	231	DFS
Seitlinger et al.	2021	Europe	R	2027	NSCLC	I‐III	32% adjuvant CT; 2% adjuvant RT; 7% adjuvant CRT	69	NLR	4.07	NR	OS

Abbreviations: AD, adenocarcinoma; CRT, chemo/radiotherapy; CT, chemotherapy; DFS, disease‐free survival; NLR, neutrophil‐lymphocyte ratio; NP, not provided; NR, not reported; NSCLC, non‐small cell lung cancer; OS, overall survival; P, prospective study; PLR, platelet‐lymphocyte ratio; R, retrospective study; RT, radiotherapy; SCC, squamous cell carcinoma.

^a^ The exact means of treatment (CT, RT, or CRT) are not mentioned.

### 3.2 NLR's impact on DFS

**FIGURE 2:**
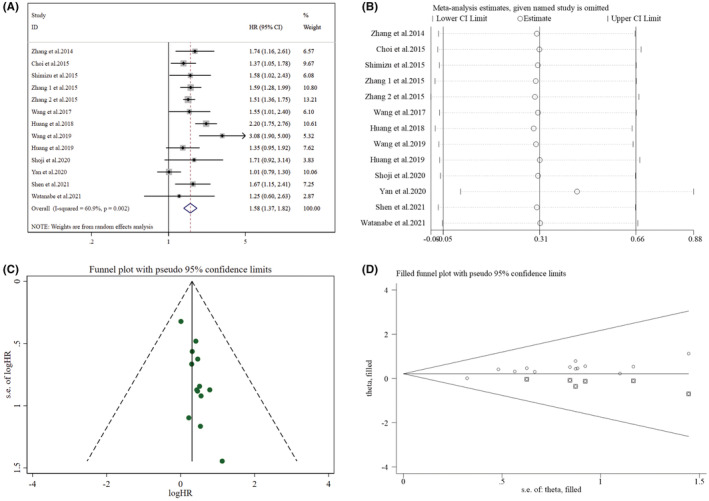
Pooled analyses of the association between preoperative NLR and DFS in resected early‐stage NSCLC patients. (A) Forest plot of the correlation between preoperative NLR and DFS. (B) Sensitivity analysis for DFS after excluding each study. (C) Funnel plot of publication bias regarding DFS. (D) Funnel plot adjusted by the trim and fill method regarding DFS.

Of the 21 studies, 20 reported the correlation between survival results and NLR in resected early‐stage NSCLC cases. Of these, 13 studies provided information on DFS (Figure 2A), which recommended that raised NLR was correlated with poor DFS (HR = 1.58, 95% CI: 1.37–1.82, *p* < 0.001). Owing to the great heterogeneity detected (*I* ^2^ = 60.90%, *p* = 0.002), a random‐effect model was utilized to evaluate the studies. From the subgroup analyses, a strong association between raised NLR and poor DFS was noted for all subgroups, indicating the reliability of the meta‐analysis. The findings of this can be found in Table 2.

**TABLE 2:** Subgroup analyses of the association between preoperative NLR and survival outcomes

Variables	N	DFS		Heterogeneity		N	OS		Heterogeneity	
		HR (95% CI)	*p*‐value	*I* ^2^	*p*‐value		HR (95% CI)	*p*‐value	*I* ^2^	*p*‐value
Total	13	1.58(1.37,1.82)	<0.001	60.90%	0.002	17	1.51(1.33,1.72)	<0.001	68.00%	<0.001
Sample size										
<300	4	1.79(1.28,2.49)	0.001	60.60%	0.055	4	1.85(1.36,2.51)	<0.001	36.20%	0.195
≥300	9	1.44(1.15,1.79)	<0.001	63.50%	0.005	13	1.45(1.26,1.67)	<0.001	72.20%	<0.001
TNM										
I‐III	10	1.56(1.32,1.84)	<0.001	70.10%	<0.001	15	1.50(1.31,1.72)	<0.001	70.60%	<0.001
Mixed (I‐II)	3	1.70(1.33,2.19)	<0.001	0.00%	0.989	2	1.63(1.07,2.48)	0.023	56.10%	0.131
Cut‐off value										
≥2.5	6	1.48(1.27,1.73)	<0.001	0.00%	0.863	8	1.67(1.46,1.91)	<0.001	1.80%	0.416
<2.5	7	1.66(1.33,2.07)	<0.001	78.90%	<0.001	9	1.40(1.16,1.68)	<0.001	79.90%	<0.001

Abbreviations: CI, confidence interval; DFS, disease‐free survival; HR, hazard ratio; NLR, neutrophil‐lymphocyte ratio; OS, overall survival.

From sensitivity analyses (Figure 2B, Supplementary Table S2), it was conducted that a single study's omission could not statistically affect the NLR's impact on DFS. Nevertheless, after excluding the study of Yan et al. (2020), the heterogeneity was found to decrease significantly (*I* ^2^ = 39.60%, *p* = 0.077), with a pooled HR of 1.65 (95% CI: 1.47–1.86, *p* < 0.001).

### 3.3 Influence of NLR on OS

**FIGURE 3:**
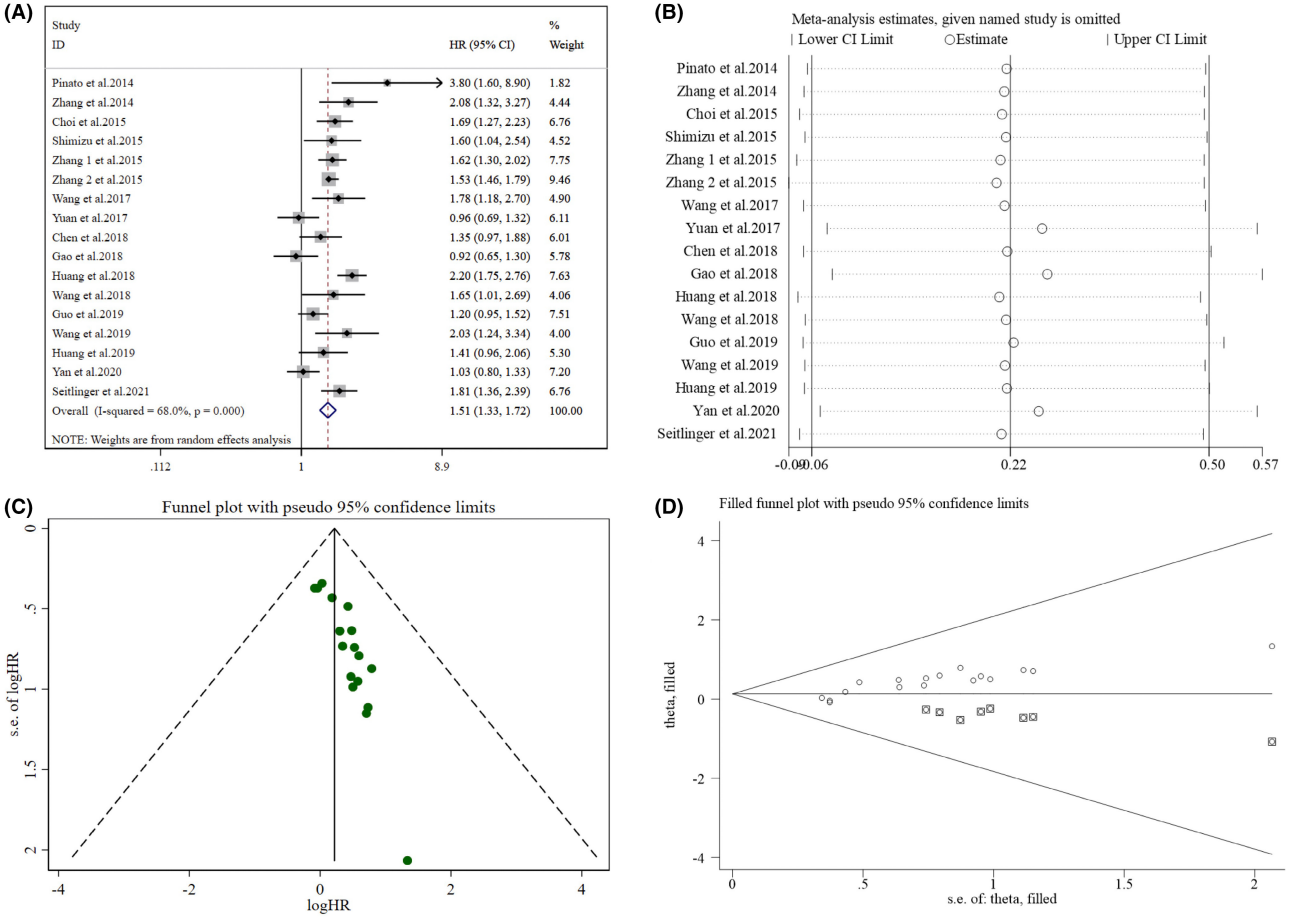
Pooled analyses of the correlation between preoperative NLR and OS in resected early‐stage NSCLC patients. (A) Forest plot of the association between preoperative NLR and OS. (B) Sensitivity analysis for OS after excluding each study. (C) Funnel plot of publication bias regarding OS. (D) Funnel plot adjusted by the trim and fill method regarding OS.

An overall of 17 studies investigated the NLR's impact on OS, among which high heterogeneity was detected (*I* ^2^ = 68.00%, *p* < 0.001). Thus, the random‐effect model was again utilized, resulting in a pooled HR of 1.51 (95% CI: 1.33–1.72, *p* < 0.001). Overall, results from these studies revealed that great NLR was linked with worse OS in cases with NSCLC (Figure 3A). This can be seen from the subgroup analyses between OS and NLR outlined in Table 2, indicating a significant association between raised NLR and poor OS.

Furthermore, sensitivity analyses exploring the potential heterogeneity of OS (Figure 3B) suggested that the omission of a single study would not be able to statistically affect the NLR's impact on OS in the meta‐analysis. In terms of OS (Supplementary Table S3), it was found that heterogeneity decreased to some extent (*I* ^2^ = 61.00%, *p =* 0.001) after excluding the study of Huang et al. (2018), with a merged HR of 1.46 (95% CI: 1.29–1.65, *p* < 0.001).

### 3.4 Effect of PLR on DFS

**FIGURE 4:**
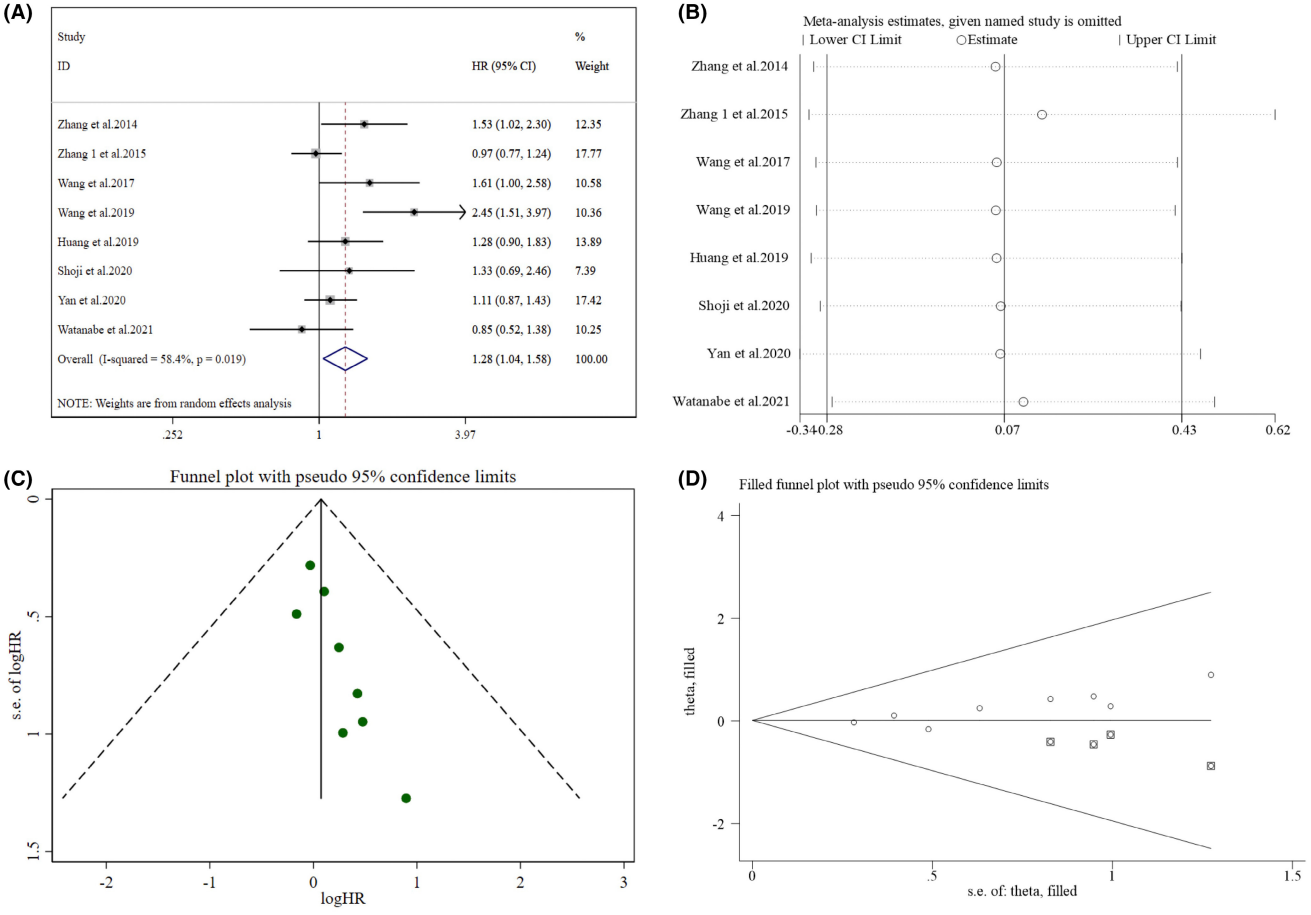
Pooled analyses of the relationship between preoperative PLR and DFS in resected early‐stage NSCLC patients. (A) Forest plot of the link between preoperative PLR and DFS. (B) Sensitivity analysis for DFS after excluding each study. (C) Funnel plot of publication bias regarding DFS. (D) Funnel plot adjusted by the trim and fill method regarding DFS.

Eight studies reported the correlation between DFS and PLR (Figure 4A) and were evaluated utilizing a random‐effect model owing to high heterogeneity (*I* ^2^ = 58.40%, *p* = 0.019). The pooled findings revealed that raised PLR was correlated with poorer DFS (HR = 1.28, 95% CI: 1.04–1.58, *p* = 0.021). As listed in Table 3, only subgroups with sample size <300 (HR = 1.67, 95% CI: 1.15–2.43, *p* = 0.008) and TNM staging of mixed (I‐II) (HR = 1.47, 95% CI: 1.04–2.07, *p* = 0.028) demonstrated a significant link between raised PLR and poor DFS.

**TABLE 3:** Subgroup analyses of the association between preoperative PLR and survival outcomes

Variables	N	DFS		Heterogeneity		N	OS		Heterogeneity	
		HR (95% CI)	*p*‐value	I^2^	*p*‐value		HR (95% CI)	*p*‐value	I^2^	*p*‐value
Total	8	1.28(1.04,1.58)	0.021	58.40%	0.019	13	1.37(1.18,1.60)	< 0.001	59.00%	0.004
Sample size										
<300	3	1.67(1.15,2.43)	0.008	55.60%	0.105	4	1.93(1.51,2.48)	< 0.001	0.00%	0.970
≥300	5	1.09(0.92,1.30)	0.308	19.70%	0.289	9	1.24(1.07,1.43)	0.004	51.10%	0.037
TNM										
I‐III	6	1.25(0.97,1.61)	0.085	67.00%	0.010	11	1.30(1.12,1.52)	0.001	55.60%	0.013
Mixed (I‐II)	2	1.47(1.04,2.07)	0.028	0.00%	0.716	2	1.87(1.37,2.54)	< 0.001	0.00%	0.695
Cut‐off value										
≥150	4	1.17(0.94,1.45)	0.168	17.60%	0.303	6	1.31(1.06,1.61)	0.012	49.40%	0.079
<150	4	1.43(0.97,2.10)	0.069	76.80%	0.005	7	1.44(1.14,1.82)	0.002	68.30%	0.004

Abbreviations: CI, confidence interval; DFS, disease‐free survival; HR, hazard ratio; OS, overall survival; PLR, platelet‐lymphocyte ratio.

In terms of DFS (Figure 4B, Supplementary Table S4), the heterogeneity decreased significantly (*I* ^2^ = 23.90%, *p* = 0.247) after ruling out the study of Wang et al. (2019), yet no association was found between preoperative PLR and DFS (HR = 1.16, 95% CI: 0.99–1.36, *p* = 0.060). Moreover, after excluding the studies of Zhang et al. (2014) (HR = 1.25, 95% CI: 0.99–1.57, *p* = 0.056) and Wang et al. (2017) (HR = 1.25, 95% CI: 1.00–1.56, *p* = 0.054), preoperative PLR and DFS remained uncorrelated.

### 3.5 PLR's influence on OS

**FIGURE 5:**
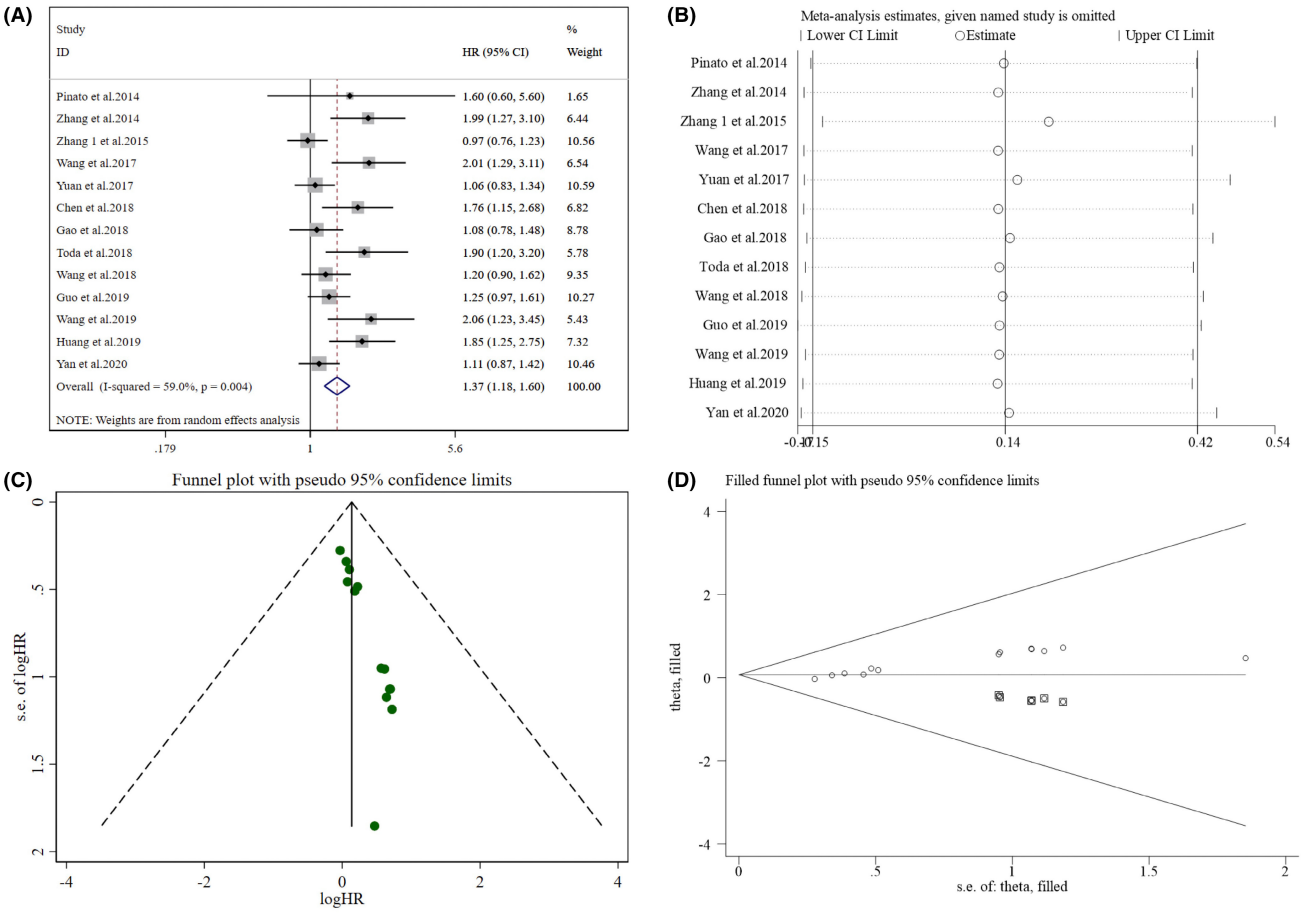
Pooled analyses of the link between preoperative PLR and OS in resected early‐stage NSCLC patients. (A) Forest plot of the relationship between preoperative PLR and OS. (B) Sensitivity analysis for OS after excluding each study. (C) Funnel plot of publication bias regarding OS. (D) Funnel plot adjusted by the trim and fill method regarding OS.

Data relating to the PLR's effect on OS was provided by 13 studies (Figure 5A). High heterogeneity (*I* ^2^ = 59.00%, *p* = 0.004) was found among these studies. Thus, a meta‐analysis was conducted utilizing the random‐effect model, from which a strong relation was found between high PLR and worse OS (HR = 1.37, 95% CI: 1.18–1.60, *p* < 0.001). For the subgroup analyses between PLR and OS (Table 3), all subgroups indicated a significant link between higher PLR and shorter OS.

As for the sensitivity analyses of OS (Figure 5B, Supplementary Table S5), after excluding the study of Zhang 1 et al. (2015), heterogeneity decreased to some extent (I^2^ = 53.50%, *p* = 0.014), with a combined HR of 1.43 (95% CI: 1.22–1.66, *p* < 0.001), suggesting that raised PLR was still linked with poor OS.

### 3.6 Publication bias

Funnel plots were found not visually asymmetric as per the influence of NLR on DFS (Figure 2C) and OS (Figure 3C), indicating a significant risk of publication bias. This observation was further validated by the findings of Egger's test for DFS (*p =* 0.001) and OS (*p* < 0.001). Subsequently, trim and fill methods were done to explore the publication bias's impact on effect estimates. No statistically significant alterations were noticed in the findings (Figures 2D and 3D). As per the PLR's influence on DFS (Figure 4C) and OS (Figure 5C), the *p* values for DFS (Egger's test, *p* = 0.012) and OS (Egger's test, *p* < 0.001) showed significant publication bias, accommodated by funnel plots which were not asymmetrical from visual inspection. Moreover, trim and fill methods again indicated no statistically significant changes in the results (Figures 4D and 5D).

## 4 DISCUSSION

Surgery has continuity as the radical medication strategy preferred for cases with early‐stage NSCLC. It is also essential in the comprehensive modality of several cases with advanced‐stage NSCLC.^34^ In recent decades, several independent prognostic biomarkers have been discovered in patients with resected NSCLC.^35^, ^36^, ^37^ Previous studies have demonstrated that NLR and PLR, as host inflammatory markers, are often used as valuable prognostic indicators for some cancers.^38^, ^39^, ^40^, ^41^ However, it remains unclear if NLR and PLR still have a similar effect in cases with resected NSCLC.

In the present study, we merged 20 NLR studies with 13,915 lung cancer cases and 15 PLR studies with 7484 lung cancer cases to further find out the NLR and PLR's prognostic impact in resected early‐stage cases with NSCLC. The meta‐analysis found that increased preoperative NLR in peripheral blood was correlated with poor DFS and OS. Despite high heterogeneity, the prognostic effect of NLR was not weakened by all subgroup analyses dependent on sample size, TNM, and cut‐off value, which supports the reliability of the meta‐analysis. Our combined findings regarding PLR still showed that high PLR was connected to poor DFS and OS. In terms of OS, each subgroup revealed a connection between longer OS and higher PLR. For the subgroup analyses between PLR and DFS, only subgroups with sample size <300 and TNM staging of mixed (I‐II) indicated a significant correlation between raised PLR and poor DFS. These results suggest that the pooled results between PLR and DFS lack stability, which may be attributed to the limited studies in the meta‐analysis.

The underlying mechanisms of the link between great preoperative NLR/PLR and poor survival results in NSCLC cases remain unknown. It has been noticed that tumor‐linked neutrophils can produce IL‐17A, promoting epithelial‐mesenchymal transition (EMT) in gastric cancer via JAK2/STAT3 signaling cascade.^42^ Additionally, the study by Mishalian et al. (2014) demonstrated that neutrophils promote regulatory T‐cells into the tumor by secreting CCL17. Regulatory T‐cells possess the function of immunosuppression and thus impair anti‐tumor immune function.^43^ Furthermore, inflammatory neutrophils can promote tumor angiogenesis and tumor progression by releasing vascular endothelial growth factor (VEGF) and Gelatinase A (MMP‐2).^44^, ^45^ Thus, raised neutrophils are often correlated with poor prognosis in cancer cases. Several studies have shown that absolute lymphocytes can anticipate long‐term survival outcomes in cancer patients.^46^, ^47^ Consequently, lymphocytes play an essential role in immune defense, and reduced lymphocytes are often associated with poorer survival in patients. Platelets were also often associated with biological processes that allowed tumors to escape immune defense, thereby promoting tumor progression and dissemination.^48^ It may therefore be concluded that NLR and PLR act essential functions in the inflammatory and anti‐inflammatory balance in the human immune response.

As a literature‐dependent meta‐analysis, several restrictions of this meta‐analysis should be noted. First, most of the literature involved were retrospective studies; hence, selection bias is present. More prospective studies are therefore required to affirm our findings. Second, only literature published in English was included in our study, with studies in other languages and unpublished studies not included, which reduced the data available for analysis. It is also possible that unpublished studies contain results considered ‘unfavorable’ or ‘negative’ by the researcher or the publisher, which may otherwise have contributed to this meta‐analysis. Third, characteristics such as sample size, region, and TNM differed throughout studies, which may also be the source of high heterogeneity. Fourth, we cannot rule out the possibility that non‐tumor‐related factors influence the blood markers of patients. Moreover, 19 out of 21 studies did not specify the pathological types of NSCLC and we could not perform subgroup analyses based on pathological type. Finally, there was a lack of standardization of cut‐off values for NLR or PLR in the included studies in the meta‐analysis. The cut‐off values of NLR (range: 1.5–5.0) and PLR (range: 81.07–300) differed throughout the involved studies. These cut‐off values were defined in different ways. For instance, in the study of Yuan et al. (2017), the appropriate cut‐off values for NLR and PLR were termed by X‐tile software.^20^ In contrast, in the study of Chen et al. (2018), the optimal values of PLR and LMR were determined by receiver operating characteristic (ROC) curve analysis.^21^ Therefore, the lack of standardization of NLR and PLR cut‐off values limits the translation of study results to clinical application. It was necessary to standardize the cut‐off values of preoperative NLR and PLR.

## 5 CONCLUSIONS

This meta‐analysis concludes that poor DFS and OS in resected early‐stage cases of NSCLC are strongly correlated with preoperative peripheral blood high NLR or PLR. This suggests that levels of NLR and PLR may serve as prognostic biomarkers in the clinic and are valuable measures for guiding patients' postoperative adjuvant treatment. In the future, more prospective, multi‐center, and large‐sample studies are needed to affirm the findings of this meta‐analysis and promote its clinical application, especially in combination with other prognostic biomarkers.

## AUTHOR CONTRIBUTIONS

The authors all participated in designing the meta analysis. Weibo Cao, Shuai Zhu, and Ning Zhou participated in the literature search. Weibo Cao and Haochuan Yu independently extrated relevant data and evaluated the quality of the studies. Tong Li, Fan Ren, and Xi Lei performed the statistical analysis. Weibo Cao wrote the first draft. Song Xu and Lingling Zu revised the article. All authors agreed on the final version of the manuscript.

## FUNDING INFORMATION

The present study was funded by the National Natural Science Foundation of China (82172776), Tianjin Science and Technology Plan Project (19ZXDBSY00060) and (303078100412), Tianjin Key Medical Discipline (Specialty) Construction Project (TJYXZDXK‐061B), and Diversified Input Project of Tianjin National Natural Science Foundation (21JCYBJC01770).

## CONFLICT OF INTEREST

The authors declare no conflict of interest.

## ETHICS STATEMENT

Ethical approval doesn't apply in the meta‐analysis.

## Supporting information


Text S1. The specific retrieval strategy in each database.

Table S1. Quality assessment by the Newcastle‐Ottawa Scale.

Table S2. Sensitivity analysis of the relationship between NLR and DFS.

Table S3. Sensitivity analysis of the association between NLR and OS.

Table S4. Sensitivity analysis of the link between PLR and DFS.

Table S5. Sensitivity analysis of the correlation between PLR and OS.

## Data Availability

All data generated or analyzed during the meta‐analysis are included in the published article and its supplementary materials.
