# Supplementary material for: Clinical significance of preoperative neutrophil‐lymphocyte ratio and platelet‐lymphocyte ratio in the prognosis of resected early‐stage patients with non‐small cell lung cancer: A meta‐analysis
Source: Cancer Med. 2022 Dec 8;12(6):7065–76. doi: 10.1002/cam4.5505 (PMC10067053; doi:10.1002/cam4.5505)
Supplement: Supplementary file 1 — Text S1. The specific retrieval strategy in each database. [file CAM4-12-7065-s006.docx]

**Supplementary Text S1.** The specific retrieval strategy in each database.

1. **PubMed retrieval strategy**

("Lung Neoplasms"[Mesh] OR "pulmonary neoplasms" OR "lung cancer" OR "Carcinoma, Non-Small-Cell Lung" OR "non-small cell lung cancer" OR "NSCLC") AND ("Surgical Procedures, Operative"[Mesh] OR "surgery" OR "operative therapy" OR "operation" OR "operative procedures" OR "invasive procedures") AND (("neutrophil lymphocyte ratio" OR "neutrophil-to-lymphocyte ratio" OR "NLR") OR ("platelet lymphocyte ratio" OR "platelet-to-lymphocyte ratio" OR "PLR")).

1. **Embase retrieval strategy**

('lung neoplasms' OR 'pulmonary neoplasms' OR 'lung cancer' OR 'carcinoma, non-small-cell lung' OR 'non-small cell lung cancer' OR 'nsclc') AND ('surgery'/exp OR 'surgical procedures, operative' OR 'operative therapy' OR 'operation' OR 'operative procedures' OR 'invasive procedures') AND (('neutrophil lymphocyte ratio'/exp OR 'neutrophil-to-lymphocyte ratio' OR 'nlr') OR ('platelet lymphocyte ratio'/exp OR 'platelet-to-lymphocyte ratio' OR 'plr')).

1. **Cochrane Library retrieval strategy**

("Lung Neoplasms" OR "pulmonary neoplasms" OR "lung cancer" OR "Carcinoma, Non-Small-Cell Lung" OR "non-small cell lung cancer" OR "NSCLC") AND ("Surgical Procedures, Operative" OR "surgery" OR "operative therapy" OR "operation" OR "operative procedures" OR "invasive procedures") AND (("neutrophil lymphocyte ratio" OR "neutrophil-to-lymphocyte ratio" OR "NLR") OR ("platelet lymphocyte ratio" OR "platelet-to-lymphocyte ratio" OR "PLR")).

1. **Web of Science retrieval strategy**

("Lung Neoplasms" OR "pulmonary neoplasms" OR "lung cancer" OR "Carcinoma, Non-Small-Cell Lung" OR "non-small cell lung cancer" OR "NSCLC") AND ("Surgical Procedures, Operative" OR "surgery" OR "operative therapy" OR "operation" OR "operative procedures" OR "invasive procedures") AND (("neutrophil lymphocyte ratio" OR "neutrophil-to-lymphocyte ratio" OR "NLR") OR ("platelet lymphocyte ratio" OR "platelet-to-lymphocyte ratio" OR "PLR")).
