# Supplementary material for: Clinical significance of preoperative neutrophil‐lymphocyte ratio and platelet‐lymphocyte ratio in the prognosis of resected early‐stage patients with non‐small cell lung cancer: A meta‐analysis
Source: Cancer Med. 2022 Dec 8;12(6):7065–76. doi: 10.1002/cam4.5505 (PMC10067053; doi:10.1002/cam4.5505)
Supplement: Supplementary file 2 — Table S1. Quality assessment by the Newcastle‐Ottawa Scale. [file CAM4-12-7065-s002.docx]

**Supplementary Table S1.** Quality assessment by the Newcastle-Ottawa Scale.

| **Author** | **Year** | **Patient selection** | **Comparability** | **Outcome assessment** | **Total scores** |
| --- | --- | --- | --- | --- | --- |
| Pinato et al. | 2014 | **** | ** | *** | 9 |
| Zhang et al. | 2014 | *** | ** | *** | 8 |
| Choi et al. | 2015 | *** | ** | *** | 8 |
| Shimizu et al. | 2015 | *** | * | *** | 7 |
| Zhang 1 et al. | 2015 | ** | ** | *** | 7 |
| Zhang 2 et al. | 2015 | *** | ** | ** | 7 |
| Wang et al. | 2017 | ** | ** | *** | 7 |
| Yuan et al. | 2017 | *** | * | *** | 7 |
| Chen et al. | 2018 | *** | ** | *** | 8 |
| Gao et al. | 2018 | ** | ** | *** | 7 |
| Huang et al. | 2018 | *** | ** | *** | 8 |
| Toda et al. | 2018 | *** | ** | ** | 7 |
| Wang et al. | 2018 | *** | ** | *** | 8 |
| Guo et al. | 2019 | *** | * | *** | 7 |
| Wang et al. | 2019 | *** | ** | ** | 7 |
| Huang et al. | 2019 | ** | ** | ** | 6 |
| Shoji et al. | 2020 | *** | * | ** | 6 |
| Yan et al. | 2020 | *** | ** | ** | 7 |
| Shen et al. | 2021 | **** | ** | *** | 9 |
| Watanabe et al. | 2021 | ** | ** | *** | 7 |
| Seitlinger et al. | 2021 | *** | ** | *** | 8 |
