# Supplementary material for: Clinical significance of preoperative neutrophil‐lymphocyte ratio and platelet‐lymphocyte ratio in the prognosis of resected early‐stage patients with non‐small cell lung cancer: A meta‐analysis
Source: Cancer Med. 2022 Dec 8;12(6):7065–76. doi: 10.1002/cam4.5505 (PMC10067053; doi:10.1002/cam4.5505)
Supplement: Supplementary file 3 — Table S2. Sensitivity analysis of the relationship between NLR and DFS. [file CAM4-12-7065-s001.docx]

**Supplementary Table S2.** Sensitivity analysis of the relationship between NLR and DFS.

| **Study omitted** | **HR (95% CI)** | ***P*-value** | **I^2^** | ***P*_H_** |
| --- | --- | --- | --- | --- |
| Zhang et al., 2014 | 1.57(1.35,1.82) | < 0.001 | 63.80% | 0.001 |
| Choi et al., 2015 | 1.61(1.38,1.87) | < 0.001 | 63.10% | 0.002 |
| Shimizu et al., 2015 | 1.58(1.36,1.83) | < 0.001 | 64.20% | 0.001 |
| Zhang 1 et al., 2015 | 1.58(1.35,1.85) | < 0.001 | 64.10% | 0.001 |
| Zhang 2 et al., 2015 | 1.60(1.35,1.89) | < 0.001 | 63.90% | 0.001 |
| Wang et al., 2017 | 1.58(1.37,1.84) | < 0.001 | 64.20% | 0.001 |
| Huang et al., 2018 | 1.51(1.33,1.71) | < 0.001 | 46.40% | 0.039 |
| Wang et al., 2019 | 1.52(1.34,1.73) | < 0.001 | 51.70% | 0.019 |
| Huang et al., 2019 | 1.60(1.38,1.86) | < 0.001 | 63.50% | 0.002 |
| Shoji et al., 2020 | 1.58(1.36,1.82) | < 0.001 | 64.10% | 0.001 |
| Yan et al., 2020 | 1.65(1.47,1.86) | < 0.001 | 39.60% | 0.077 |
| Shen et al., 2021 | 1.57(1.36,1.83) | < 0.001 | 64.00% | 0.001 |
| Watanabe et al., 2021 | 1.59(1.38,1.84) | < 0.001 | 63.80% | 0.001 |

Abbreviations: NLR, neutrophil-lymphocyte ratio; DFS, disease-free survival; HR, hazard ratio; CI, confidence interval; *P*_H_, *P-*value for heterogeneity.
