# Supplementary material for: Clinical significance of preoperative neutrophil‐lymphocyte ratio and platelet‐lymphocyte ratio in the prognosis of resected early‐stage patients with non‐small cell lung cancer: A meta‐analysis
Source: Cancer Med. 2022 Dec 8;12(6):7065–76. doi: 10.1002/cam4.5505 (PMC10067053; doi:10.1002/cam4.5505)
Supplement: Supplementary file 4 — Table S3. Sensitivity analysis of the association between NLR and OS. [file CAM4-12-7065-s004.docx]

**Supplementary Table S3.** Sensitivity analysis of the association between NLR and OS.

| **Study omitted** | **HR (95% CI)** | ***P*-value** | **I^2^** | ***P*_H_** |
| --- | --- | --- | --- | --- |
| Pinato et al., 2014 | 1.49(1.31,1.68) | < 0.001 | 67.00% | < 0.001 |
| Zhang et al., 2014 | 1.49(1.31,1.70) | < 0.001 | 68.70% | < 0.001 |
| Choi et al., 2015 | 1.50(1.31,1.72) | < 0.001 | 69.60% | < 0.001 |
| Shimizu et al., 2015 | 1.51(1.32,1.72) | < 0.001 | 69.90% | < 0.001 |
| Zhang 1 et al., 2015 | 1.51(1.31,1.73) | < 0.001 | 69.70% | < 0.001 |
| Zhang 2 et al., 2015 | 1.52(1.30,1.77) | < 0.001 | 69.90% | < 0.001 |
| Wang et al., 2017 | 1.50(1.31,1.71) | < 0.001 | 69.60% | < 0.001 |
| Yuan et al., 2017 | 1.55(1.37,1.76) | < 0.001 | 64.50% | < 0.001 |
| Chen et al., 2018 | 1.52(1.33,1.74) | < 0.001 | 69.70% | < 0.001 |
| Gao et al., 2018 | 1.55(1.37,1.76) | < 0.001 | 64.20% | < 0.001 |
| Huang et al., 2018 | 1.46(1.29,1.65) | < 0.001 | 61.00% | 0.001 |
| Wang et al., 2018 | 1.51(1.32,1.72) | < 0.001 | 69.90% | < 0.001 |
| Guo et al., 2019 | 1.54(1.35,1.76) | < 0.001 | 67.50% | < 0.001 |
| Wang et al., 2019 | 1.49(1.31,1.70) | < 0.001 | 69.10% | < 0.001 |
| Huang et al., 2019 | 1.52(1.33,1.74) | < 0.001 | 69.90% | < 0.001 |
| Yan et al., 2020 | 1.55(1.37,1.76) | < 0.001 | 63.20% | < 0.001 |
| Seitlinger et al., 2021 | 1.49(1.30,1.71) | < 0.001 | 68.90% | < 0.001 |

Abbreviations: NLR, neutrophil-lymphocyte ratio; OS, overall survival; HR, hazard ratio; CI, confidence interval; *P*_H_, *P-*value for heterogeneity.
