# Supplementary material for: Clinical significance of preoperative neutrophil‐lymphocyte ratio and platelet‐lymphocyte ratio in the prognosis of resected early‐stage patients with non‐small cell lung cancer: A meta‐analysis
Source: Cancer Med. 2022 Dec 8;12(6):7065–76. doi: 10.1002/cam4.5505 (PMC10067053; doi:10.1002/cam4.5505)
Supplement: Supplementary file 5 — Table S4. Sensitivity analysis of the link between PLR and DFS. [file CAM4-12-7065-s003.docx]

**Supplementary Table S4.** Sensitivity analysis of the link between PLR and DFS.

| **Study omitted** | **HR (95% CI)** | ***P*-value** | **I^2^** | ***P* _H_** |
| --- | --- | --- | --- | --- |
| Zhang et al., 2014 | 1.25(0.99,1.57) | 0.056 | 60.80% | 0.018 |
| Zhang 1 et al., 2015 | 1.36(1.08,1.70) | 0.008 | 51.90% | 0.052 |
| Wang et al., 2017 | 1.25(1.00,1.56) | 0.054 | 60.70% | 0.018 |
| Wang et al., 2019 | 1.16(0.99,1.36) | 0.060 | 23.90% | 0.247 |
| Huang et al., 2019 | 1.29(1.01,1.65) | 0.042 | 64.00% | 0.011 |
| Shoji et al., 2020 | 1.28(1.02,1.61) | 0.003 | 64.10% | 0.010 |
| Yan et al., 2020 | 1.33(1.03,1.72) | 0.031 | 63.10% | 0.012 |
| Watanabe et al., 2021 | 1.34(1.08,1.67) | 0.009 | 59.30% | 0.022 |

Abbreviations: PLR, platelet-lymphocyte ratio; DFS, disease-free survival; HR, hazard ratio; CI, confidence interval; *P*_H_, *P-*value for heterogeneity.
