# Supplementary material for: Clinical significance of preoperative neutrophil‐lymphocyte ratio and platelet‐lymphocyte ratio in the prognosis of resected early‐stage patients with non‐small cell lung cancer: A meta‐analysis
Source: Cancer Med. 2022 Dec 8;12(6):7065–76. doi: 10.1002/cam4.5505 (PMC10067053; doi:10.1002/cam4.5505)
Supplement: Supplementary file 6 — Table S5. Sensitivity analysis of the correlation between PLR and OS. [file CAM4-12-7065-s005.docx]

**Supplementary Table S5.** Sensitivity analysis of the correlation between PLR and OS.

| **Study omitted** | **HR (95% CI)** | ***P*-value** | **I^2^** | ***P* _H_** |
| --- | --- | --- | --- | --- |
| Pinato et al., 2014 | 1.37(1.17,1.61) | < 0.001 | 62.20% | 0.002 |
| Zhang et al., 2014 | 1.33(1.15,1.55) | < 0.001 | 56.40% | 0.008 |
| Zhang 1 et al., 2015 | 1.43(1.22,1.66) | < 0.001 | 53.50% | 0.014 |
| Wang et al., 2017 | 1.33(1.15,1.55) | < 0.001 | 55.90% | 0.009 |
| Yuan et al., 2017 | 1.42(1.21,1.68) | < 0.001 | 58.80% | 0.005 |
| Chen et al., 2018 | 1.35(1.15,1.58) | < 0.001 | 59.10% | 0.005 |
| Gao et al., 2018 | 1.41(1.20,1.66) | < 0.001 | 61.00% | 0.003 |
| Toda et al., 2018 | 1.35(1.15,1.57) | < 0.001 | 58.70% | 0.005 |
| Wang et al., 2018 | 1.40(1.19,1.66) | < 0.001 | 62.20% | 0.002 |
| Guo et al., 2019 | 1.40(1.18,1.66) | < 0.001 | 62.40% | 0.002 |
| Wang et al., 2019 | 1.34(1.15,1.56) | < 0.001 | 57.40% | 0.007 |
| Huang et al., 2019 | 1.34(1.15,1.56) | < 0.001 | 57.00% | 0.007 |
| Yan et al., 2020 | 1.42(1.20,1.68) | < 0.001 | 60.60% | 0.003 |

Abbreviations: PLR, platelet-lymphocyte ratio; OS, overall survival; HR, hazard ratio; CI, confidence interval; *P*_H_, *P-*value for heterogeneity.
